# Tetanus Treated by Injections of Antitoxin into the Spinal Theca

**Published:** 1904-03-19

**Authors:** 


### TETANUS TREATED BY INJECTIONS OF ANTITOXIN INTO THE SPINAL THECA.

Wallace and Sargant,[1] writing on the above subject, describe three successful and one unsuccessful case. Antitetanic serum was injected into the spinal cord in the lumbar region after some of the cerebro-spinal fluid had been withdrawn. The first case was a woman aged 43 who had become infected through a cut head 17 days before treatment and nine days before the first symptom. The first general convulsion took place as chloroform was being administered. The wound was excised and 10 c.c. of serum injected into the lumbar spinal theca. No further injection was made, but chloral and bromide were administered and the patient was fed per rectum : she recovered on the 24th day. The second case was a man aged 38 who had infected himself through a torn nail 17 days before treatment and 11 days before the first symptom. Chloroform was administered, the finger amputated, and serum injected. Thirty cubic centimetres were used during 21 hours in three injections. There was one convulsion after the first injection. Bromide and chloral were exhibited. Gradually the spasm passed off, and patient was discharged on the 28th day. Case three was a man of 31 who had run a splinter beneath his nail while gardening four days before treatment and three days before the first symptom. The case was one of acute tetanus, and he had a convulsive attack at the commencement of the induction of chloroform anæsthesia. The finger was amputated, and 10 c.c. of serum injected into the spinal theca on two occasions. At first the symptoms progressed, but from the sixth day they gradually diminished in severity ; and though he suffered from delirium and respiratory embarrassment for 26 days, he ultimately recovered about the 42nd day. Chloral and bromide were administered during the first 12 days.

The fourth case was that of a man aged 42 years. Six days before treatment he wounded his foot with a pickaxe. He was treated on the first day of disease. There was no general convulsion until chloroform was administered. The wound had been dressed at the time it was inflicted. After excision it was swabbed out with 10 in 11 carbolic acid. Twenty cubic centimetres of serum were injected, and this dose repeated in 12 hours. The patient died eight hours after the second injection. There is apparently no ill effect from this mode of administering antitetanic serum, and it seems more effectual than the subcutaneous and less difficult and dangerous than the intracranial method.

[1] Lancet, March 5th, 1904.

